# A Digital Platform and Smartphone App to Increase Physical Activity in Patients With Type 2 Diabetes: Overview Of a Technical Solution

**DOI:** 10.2196/40285

**Published:** 2023-03-14

**Authors:** Stephanie E Bonn, Christina Alexandrou, Ylva Trolle Lagerros

**Affiliations:** 1 Clinical Epidemiology Division Department of Medicine Solna Karolinska Institutet Stockholm Sweden; 2 Department of Biosciences and Nutrition Karolinska Institutet Stockholm Sweden; 3 Division of Society and Health Department of Health, Medicine and Caring Sciences Linköping University Linköping Sweden; 4 Obesity Specialist Center Academic Specialist Center Stockholm Health Services Stockholm Sweden

**Keywords:** methods, mHealth, mobile app, self-management, smartphone, digital, platform, physical activity, diabetes, technical, engagement, self-care, development, app, walking, effective

## Abstract

Mobile Health (mHealth) solutions can be used to increase patients’ engagement in self-care. Descriptions of the technical solutions behind mHealth smartphone apps may guide the development of future apps. Here, we aim to describe the technical background and visual display of the DiaCert system, which was developed to support daily walking among patients with type 2 diabetes. The DiaCert system publishes one application program interface developed for patient devices (ie, smartphone apps running on iOS or Android) and another for web-based health care provider components (ie, administrative components). An individual care plan is created for each patient on the caregiver platform, and data on physical activity (ie, steps), blood samples, and questionnaires are shared between patient and caregiver in the DiaCert system. Technical solutions such as this enable us to reach more individuals at a lower cost compared to traditional health care. An advantage to the DiaCert technical solution is that it is built on a simple architecture and therefore is easily scalable. However, as it is a separate solution, it means adding yet another process for health care personnel to integrate into their work, which must be acknowledged. We hope that the technical description and visual display of the DiaCert system herein can guide researchers in the design and building of new and effective mHealth solutions.

**International Registered Report Identifier (IRRID):**

RR2-10.1186/s12889-018-5026-4

## Introduction

New technology offers new opportunities for health care providers to connect, support, and monitor their patients. Traditional health care has often been organized as (1) reactive (ie, acting when there is an acute need) and (2) episodic (eg, annual checkups). However, today’s technology enables the landscape of health care to become (1) preventive and (2) continuous, that is, continuously following the health of patients and thereby enabling prevention of disease progression and complications from disease.

Type 2 diabetes is a chronic disease whose prevalence is rising rapidly [1]. Given the shortage of diabetes health professionals, especially in low- and middle-income countries, where the prevalence is rising most rapidly [2], use of new technology can be part of the solution. In addition to medication, a healthy lifestyle, including weight management, healthy eating habits, and physical activity, is key to controlling diabetes. Mobile health (mHealth) could potentially transform traditional health care toward becoming more preventive and continuous. This can increase patients’ engagement through the use of self-monitoring between routine care visits [3].

Smartphone app interventions targeting physical activity have proven effective [4]. Different apps include different features, such as monitoring, goal setting, and feedback [5]. Although detailed descriptions of app features and interventions are available, it is uncommon for researchers to publish any details about the technical solutions behind their apps, even though such information could be useful and improve the development of future apps.

The aim of this paper is to describe the technical background and provide a visual display of a smartphone app developed to support persons with type 2 diabetes and help them increase their daily physical activity. The smartphone app, in which patients register their daily steps, has been evaluated in a randomized controlled trial described in detail elsewhere [6]. We believe that the information given in this viewpoint can be used to guide and inspire future health app development.

## Methods

### Technical Solution

**Figure 1:**
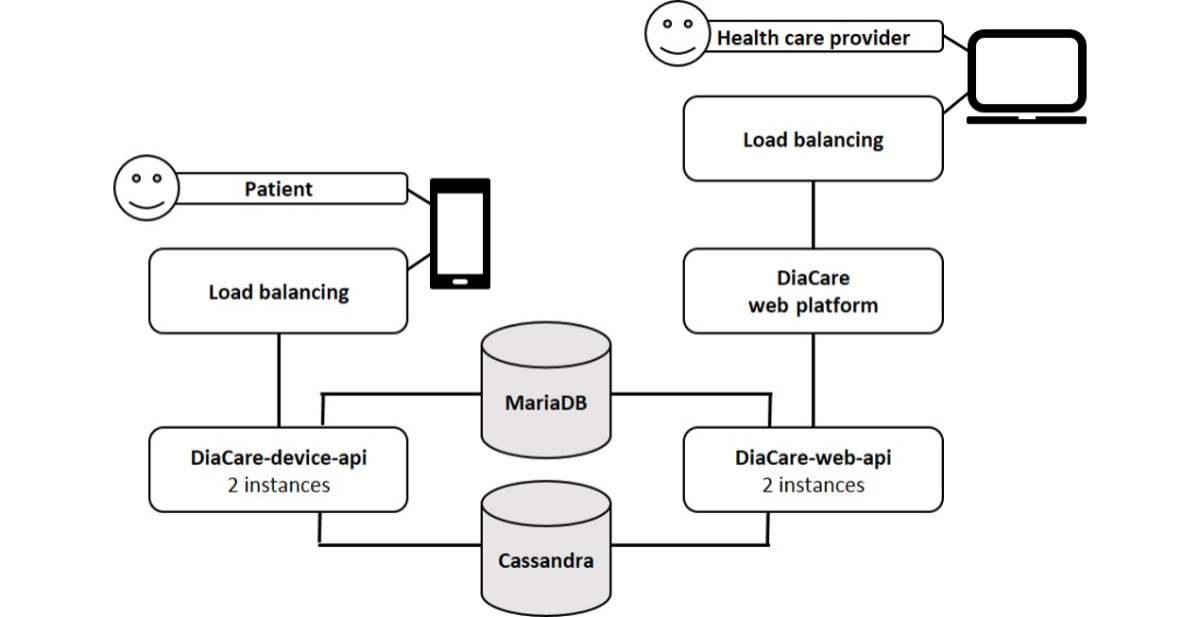
Schematic of the DiaCert system. API: application programming interface.

DiaCert comprises several systems that together create one solution to collect health data from patients. The DiaCert system publishes one application programming interface (API) developed for patient devices (ie, smartphone apps running on iOS or Android) and another for web-based health care provider components (eg, administrative components). The latter API also provides back-office functionality to include new health care providers and patients in the system. The DiaCert system uses 2 persistent databases: the MySQL MariaDB database (MariaDB Foundation) and the NoSQL Cassandra database (Apache Software Foundation). The MariaDB database is used to store information regarding users (ie, health care providers and patients) and the Cassandra database is used to store collected data (ie, steps and results from blood samples). The server applications are run in Docker containers on the application servers. The containers are run on the OpenShift container platform. Load balancers are used in order to distribute the load from the client to the server applications. Components of the DiaCert system are shown in Textbox 1 and Figure 1.

**Components and hierarchy of the DiaCert system and division of front and back ends.**

**Front end**

- Patient component
  The DiaCert iOS app
  The DiaCert Android app
- Health care provider components and administrative components (web based)

**Back end**

- Web application program interface (DiaCare-web-api)
- Device application program interface (DiaCare-device-api)
- MariaDB database
- Cassandra database

### Health Care Provider Component

An individual care plan is created for each patient entered into the caregiver platform using their unique national registration number [7]. Each caregiver has his or her own list of registered patients. A random code of 6 digits or letters and a QR code are created by the caregiver in the platform and used to connect the caregiver platform to the patient app. To ensure security, the code is only valid for 5 minutes. In the individual care plan, the patient and the caregiver set a daily step goal together. The goal is personalized, and no predefined recommendations on how to reach a set goal are used. The goal can be revised by the caregiver in dialogue with the patient. Steps are uploaded directly to the caregiver platform from the patient’s smartphone. The care plan also includes dates for laboratory testing and lifestyle questionnaires.

### Patient Component: Smartphone App

**Figure 2:**
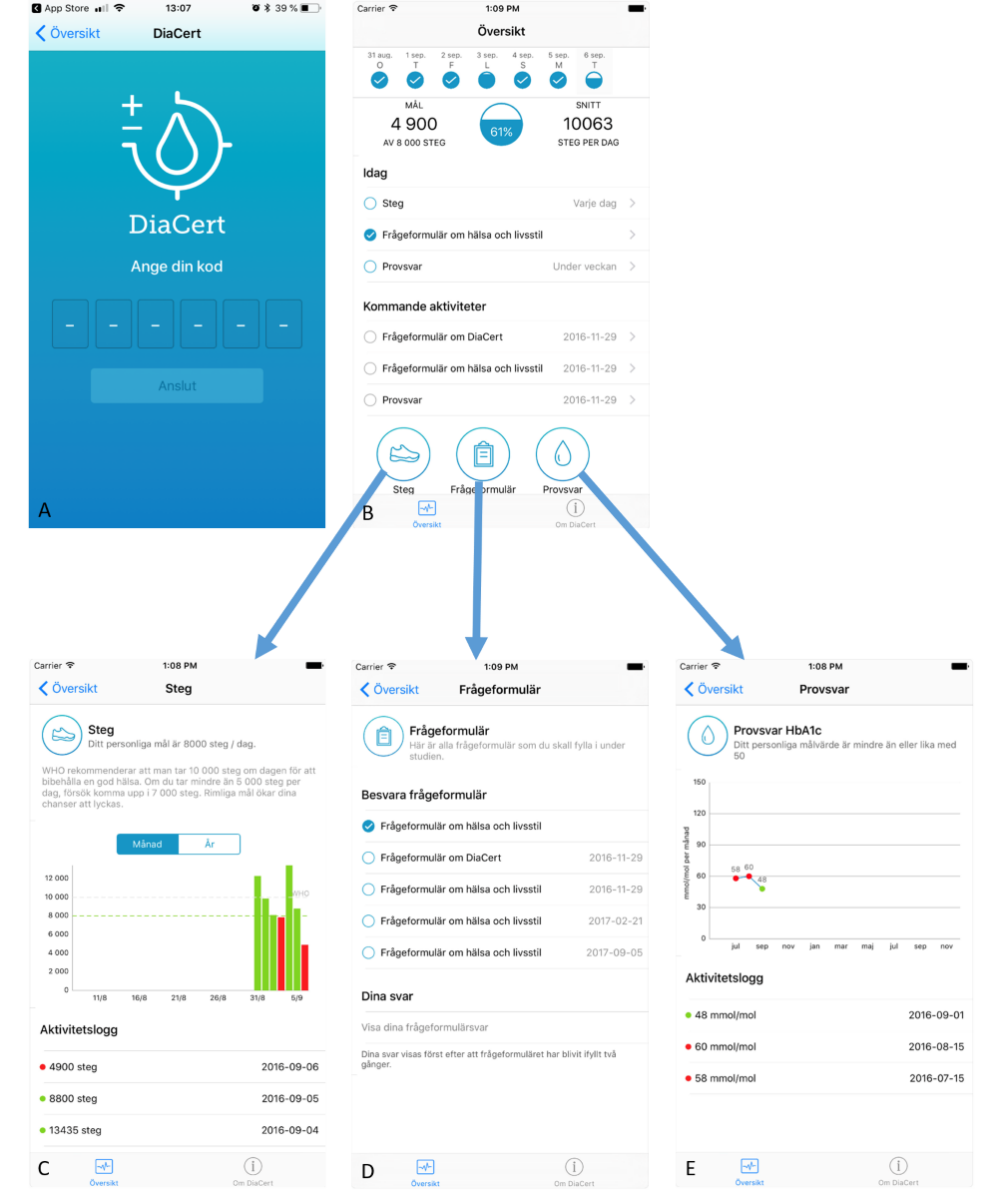
The patient smartphone app with each screen (A-E).

Figure 2 shows the patient smartphone app, screen by screen.

The patient app is connected to the health care platform using a unique code received from the caregiver (Figure 2A). The patient only needs to connect using the code once.

The home screen (Figure 2B) shows a summary of activities in the individual care plan and presents an overview of steps taken that day and the last 7 days and dates for laboratory tests and questionnaires. The individual step goal is displayed, and that day’s current number of steps is shown as a percentage of the daily goal. Circles at the top of the screen illustrate goal fulfillment during the last 7 days; below, a larger circle represents that day’s steps. The circle is gradually filled as the patient walks toward the goal. When the goal is reached, the circle is completely filled and marked with a checkmark. Below that, 3 symbols (a shoe, a clipboard, and a drop of blood) lead to detailed information on steps, questionnaires, and results from blood samples (Figure 2C-E).

Steps (Figure 2C) are displayed as a bar chart and can be shown either as an average per month or steps per day. Green bars indicate days when the step goal was reached, and red bars indicate days the goal was not reached. The next screen (Figure 2D) shows questionnaires to fill out and responses from previous questionnaires. The final screen (Figure 2E), indicated by the drop of blood, shows results from laboratory tests. The example in Figure 2E shows data on hemoglobin A_1C_ levels at 3 time points.

Eleven persons aged 19 to 49 years pilot-tested the first version of the DiaCert app for 3 weeks. Only 4 of 11 (36%) felt inspired to increase their physical activity during the test, and testers primarily requested an improved layout and better overview of their physical activity. In the next version of the app, we added the circles showing the continuous addition of steps to reach 100% of the daily goal and bar charts displaying goal fulfillment over time.

## Discussion

### Overview

Technical solutions such as the DiaCert system enable us to reach more individuals at a lower cost than with traditional health care. The popularity of mHealth apps has led to a vast number of apps being freely available on the market that target health management in chronic conditions [8,9]. Only a fraction of all available apps have published technical descriptions.

A strength of the DiaCert technical solution is that it is built on a simple architecture and is thus easily scalable. A connection with other platforms would have increased complexity and costs. Nonetheless, the lack of integration with existing information systems, such as those that document continuous glucose data and make the data available for treating nurses or physicians, is a limitation. Furthermore, as DiaCert is a separate solution, using it means adding yet another process for health care personnel to integrate into their busy schedules. Another limitation is that the front end was only developed for health care personnel and not for private carers, such as carers of patients with cognitive impairment.

Behavior change techniques that impact the effectiveness of interventions have been summarized previously by Sporrel et al [5]. The DiaCert system comprises several techniques described by Sporrel et al, including monitoring, sharing, goal setting, and positive feedback. Passive monitoring, such as automatically recording step count in a participant’s phone, limits the time and effort of the user. In DiaCert, patients share their data with the caregiver. Sharing may increase effectiveness, according to Sporrel et al, but little is known about how sharing of physical activity information between patient and caregiver impacts the activity levels of the patient. Nevertheless, many patients show willingness to share personal digital data with their health care providers [10]. Two-way communication between persons with diabetes and health care providers has been determined to be a key component in technology-based self-care interventions that successfully reduce long-term blood sugar levels [11].

Another advantage of DiaCert is that a personalized goal is set with the caregiver. Assigning a goal to users, rather than letting them set their own goal or using a static, generic goal, was shown to be advantageous in the scoping review by Sporrel at al. Rewards for goal achievement, which are included in DiaCert, were also shown to be effective. Further, the DiaCert app is visual, and the number of daily steps is easy to follow regardless of language or literacy. Thus, use of the app is independent of language. This is valuable, as type 2 diabetes is common in immigrants [12].

### Conclusions

We hope that our technical description and visual display of the DiaCert system can inspire development of future health apps and guide researchers in the design and building of new and effective mHealth solutions. Digital solutions have the potential to empower patients to manage their own health data and monitor their lifestyle to improve health.

## Abbreviations

API: application programming interface
mHealth: mobile health
